# Emplacement of screen-printed graphene oxide coating for building thermal comfort discernment

**DOI:** 10.1038/s41598-020-72670-8

**Published:** 2020-09-23

**Authors:** Anurag Roy, Aritra Ghosh, David Benson, Tapas K. Mallick, Senthilarasu Sundaram

**Affiliations:** Environment and Sustainability Institute, University of Exeter, Penryn Campus, Cornwall, TR10 9FE UK

**Keywords:** Graphene, Materials for devices, Nanoscale materials, Structural materials

## Abstract

This study demonstrates the development of flexible graphene oxide coatings (GOCs) by the screen-printed technique and further its implementation as a thermal absorber for buildings’ thermal comfort purpose. The basic concept consists the integration of the GOC as a flat absorber on the top of a low iron glass or aluminium-based substrate (5 × 5 cm^2^) connecting through a phase change material channel in contact with direct sun exposure. The function of GOC as an outdoor cover of the prototype chamber is to maintain the high indoor temperature while the outdoor temperature is low. Using the GOC, it has been observed that the indoor temperature (at the substrate) of the prototype chamber always remains higher as compared to the outdoor temperature (at the GOC) as measured under 1 SUN 1.5 AM condition. The temperature difference between outdoor and indoor exterior surface significantly increases during the light exposure time, whereas the difference drastically approaches to zero during the cooling period. The variation of different crucial environmental factors such as high temperature, moisture, flexibility and water resistivity has been investigated on the developed GOCs to understand the stability of the coating further.

## Introduction

Currently, the building consumes excessive energy due to heating and cooling load demand^1^. Temperature swing into a building interior is the root cause of the discomfort. Traditionally using sensible heat storage-based building envelope as thermal mass or inertia, this temperature fluctuation was reduced, which is not efficient. The latent heat storage medium is superior to sensible as it can delay the rise and fall of building interior temperature. The latent heat storage medium has 5–14 times more heat storage ability than the sensible heat storage medium^2^. Building in northern latitude climate (colder climate) requires the heating load to maintain the comfortable interior temperature. The phase change material (PCM) based latent heat storage can releases the stored heat after sunset, which can reduce the daily temperature swing. Up to 10° to 20° C of temperature swing, swing reduction is possible by using this technique^3–7^. The PCM operation is restricted mainly due to its low thermal conductivity and leakage issue during phase transition. The heat transfer rate has been suppressed due to the low thermal conductivity of PCM^8–11^. At the same time, during the phase transition stage, the leakage of PCM affects the system, which limits its further use. However, this low thermal conductivity of PCM, which can be improved by using nanoparticle incorporation^12,13^. In this regard, high conductive nanoparticle dispersion could be a better alternative solution of PCM to enhance its thermal conductivity^14,15^. Though, factors such as surface-functionalization, surface area, shape and size of nanoparticle are also controlled the efficacy of the PCM operation^16,17^. However, nanoparticle addition does not affect the water-resistant property of PCM.

Further, the preparation and fabrication process of nanoparticle incorporation in PCM becomes quite inflexible for its on-site application too. Placement of reflecting and absorbing coating plays a crucial role in building interior comfort. While for summer climate reflecting coating at the exterior building wall reduce the solar transmission reflecting coating at the building interior is suitable for the colder climate where the long wavelength radiation inside the building get reflected from the wall and stay stored inside a building. Solar absorbing coating at the external wall of the building allows more heat to penetrate inside a building which offers comfort temperature and suitable for colder climate building^18,19^.

The solar absorbing coating can utilize solar thermal energy appropriately^20–22^. In recent years, considerable efforts have been executed to develop and prepare novel, efficient and excellent weather resistance coatings worldwide. Many novels, efficient solar absorber coatings are developed in recent decades^23–25^. However, the methods of qualification tests, such as the test of thermal stability, corrosion resistance and prediction of service life are still the critical challenges for solar absorber coating to be applied for large-scale applications. At this scenario, light-weighted, flexible, and of course, higher specific heat capacity and a better thermal conductive material is required. With a relatively simple generation process and enhanced optical absorption, carbon materials will help to expand the application of graphene-based materials as broadband solar absorbers. The Dirac fermions (zero effective mass) of graphene provides high electron mobilities up to 200 k cm^2^V^−1^ s^−1^^26^. The combination of high broadband absorption up to 85% (~ 1200 nm), high specific surface area (> 2000 m^2^ g^−1^), excellent mechanical properties such as high Young’s modulus (1.1 TPa) and excellent thermal stability (> 200 °C) compare to many polymers makes the carbon as an effective photo-thermal conversion layer in the interfacial evaporation system^27,28^. A perfect monolayer of graphene can absorb ∼ 2.3% incident solar light in the visible range along with excellent electrical and thermal conductivity^29,30^. Having high electrical-thermal conductivity and excellent water-resistive features coupled with flexibility in a single material, graphene and related derivatives such as graphene oxide, fibres, foams etc. has been turned up as a fantastic material for various thermal applications^31–35^. Graphene has been successfully employed as a heat-spreader candidate for power electronics, automotive electronics devices^36,37^. Besides, graphene has also been used along with the PCM for photo-to-thermal conversion study^38^. However, as a building-integrated application, the studies with graphene is relatively rare. However, the synthesis of graphene is quite expensive, restricted to many chemical reactions (need surface functionalization), and thus retard mass production for large-scale application. In that respect, graphene oxide (GO) is cheaper and easier to manufacture than graphene, and so may enter mass production and use for large-scale applications too. Besides, GO can easily be mixed with different polymers and other materials in order to enhance the properties of composite materials like tensile strength, elasticity, conductivity, and so on.

Presently, the room heater is a quite popular heating appliance worldwide. However, using the heater appliances further blemishes environment associated with the depletion of ozone level and global climate. Passive heating techniques thus elicit the attention in terms of its low energy consumption, environment-friendly degree of comfort. GO emerges as a low-cost suitable passive cooling/heating candidate for energy and environmentally friendly building thermal comfort solution^38–40^. Containing different epoxy and hydroxyl groups at the surface, GO exhibits very unique chemical, optical and electronic properties to become an independent solar absorber material^41,42^. Besides, GO has been found as a more scalable alternative to graphene for large-scale application.

Though, different ceramics, inorganic oxides, phase change materials, cermet and composite materials are already available as a trendsetter solar heat absorber and mostly their preparation and application dealing with the environmental factor, energy consumption, toxicity and of course cost-effectiveness. In terms of cost and eco-friendly sustainable material development, graphene-based materials provide new opportunities to current existing adsorbents employed for the solar heat removal coating material^43^. Despite that, the hydrophobic property of GO enables both preventing water passage and resisting damp, and water ingress to the building structure. In addition, building coatings are supposed to feel pleasant to the touch and to look good for as long as possible, be easy to maintain and not be spoiled by dirt, water stains or finger marks. Therefore, features like anti-scratching, flexibility and anti-dust properties of GO coatings further attracted as a suitable coating material in a single system for the building application^44,45^. Using PCMs go through a phase change, and accordingly, they are capable of storing thermal energy, allowing for stabilization of temperature^46–48^.

In addition, using GO on flexible substrate allows ease of storage in rolled-up form for new fields such as building-integrated photovoltaics and flexible electronic devices, Primary considerations for the selection of appropriate metal substrates are the thermal expansion coefficient, surface roughness, impurity content, and cost. Polymer films such as polyethylene terephthalate (PET) and polyethylene naphthalate (PEN) are already used as transparent and lightweight substrates for photovoltaic applications but restricted to apply for high-temperature processing. Stainless steel (StS) is considerably less expensive than its glass counterpart is, and it has the added benefit of being easy to large production through roll-to-roll fabrication. Besides, the less-explored StS is attractive for the PV industry due to excellent chemical, mechanical and thermal stability, widely used in the industry, relatively cheap, readily available in thin foils, high temperature compatible, with shallow surface roughness.

In this regard, applying graphene as a separate layer over PCM brings to light the possible underlying science under the change in the thermal properties of PCM and therefore, its efficacy. In this work, the thermal performance of the screen-printed graphene coating as a heat spreader was evaluated in terms of its integration in a 5 cm × 2 cm × 2 cm definitive prototype chamber and the corresponding temperature profile experiment has been performed under 1 SUN 1.5 A.M. Besides, the high temperature and water resistivity studies of the graphene oxide coating has been further investigated to understand its stability. It has been observed that graphene oxide coating has the potential to be a promising heat spreader material for thermal management of hot spots in buildings’ thermal comfort analysis.

## Results and discussion

### Prototype testing and passive cooling property analysis

**Figure 1:**
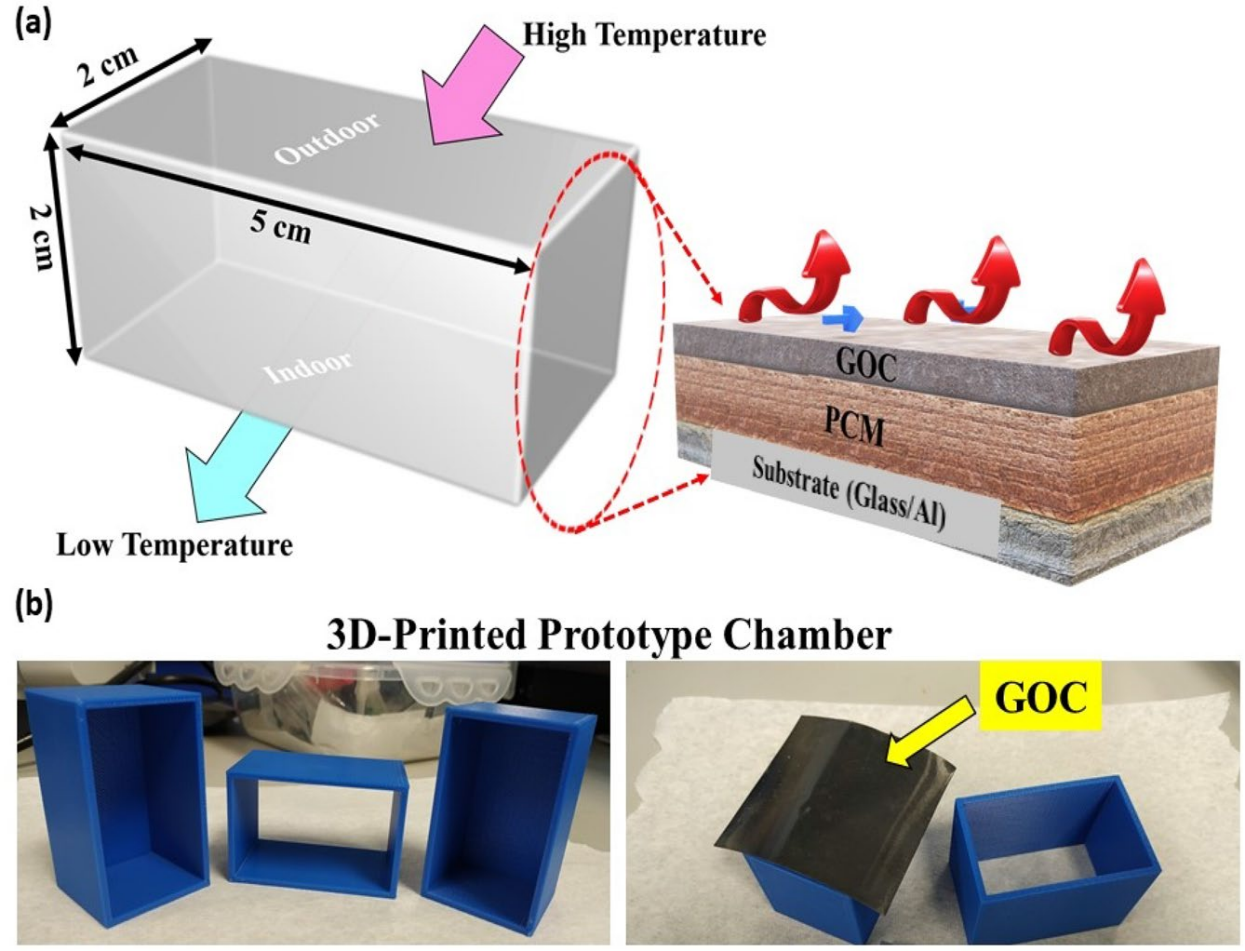
(**a**) Schematic diagram of the design and proposed experiment using a prototype chamber design and (**b**) digital image of the 3D printed prototype chamber, where GOC has been used as a top layer of the solar absorber.

In order to monitor the high-temperature resistive effect, the integration of the GOC as a flat absorber strip on the top of a glass or aluminium (Al) substrate connecting through a PCM channel was further executed in contact with direct sun exposure. Accordingly, 5 cm × 2 cm × 2 cm definitive three dimensional (3D) printed prototype chamber has been made with an aperture area of 0.48 m^2^. The GOC was placed as a shed (top), facing the coating side towards sunlight (outdoor) followed by a PCM filling and a glass-based floor. The overall prototype chamber design and a digital photograph of the fabricated system have been given in Fig. 1.

### Passive cooling experiments with GOC based prototype chamber

A comparative study of the GOC on glass and Al was further executed in order to understand the thermal resistant behaviour of the GOC on different substrates. Al sheet is considerably lightweight, flexible and secure to handle than the glass counterpart. The thermal effect in terms of the temperature difference between the two ends of the developed prototype chamber, the following sets of experiments were further designed such as.

- Glass-based GOC
  - Only Glass (Outdoor)-PCM-Only Glass (Indoor): GG
  - GOC-Glass (Outdoor)-PCM-Only Glass (Indoor): GOCGG
  - GOC-Glass (Outdoor)-PCM-Only Al (Indoor): GOCGA.
- Al-based GOC
  - Only Al (Outdoor)-PCM-Only Al (Indoor): AA
  - GOC-Al (Outdoor)-PCM-Only Glass (Indoor): GOCAG
  - GOC-Al (Outdoor)-PCM-Only Al (Indoor): GOCAA.

**Figure 2:**
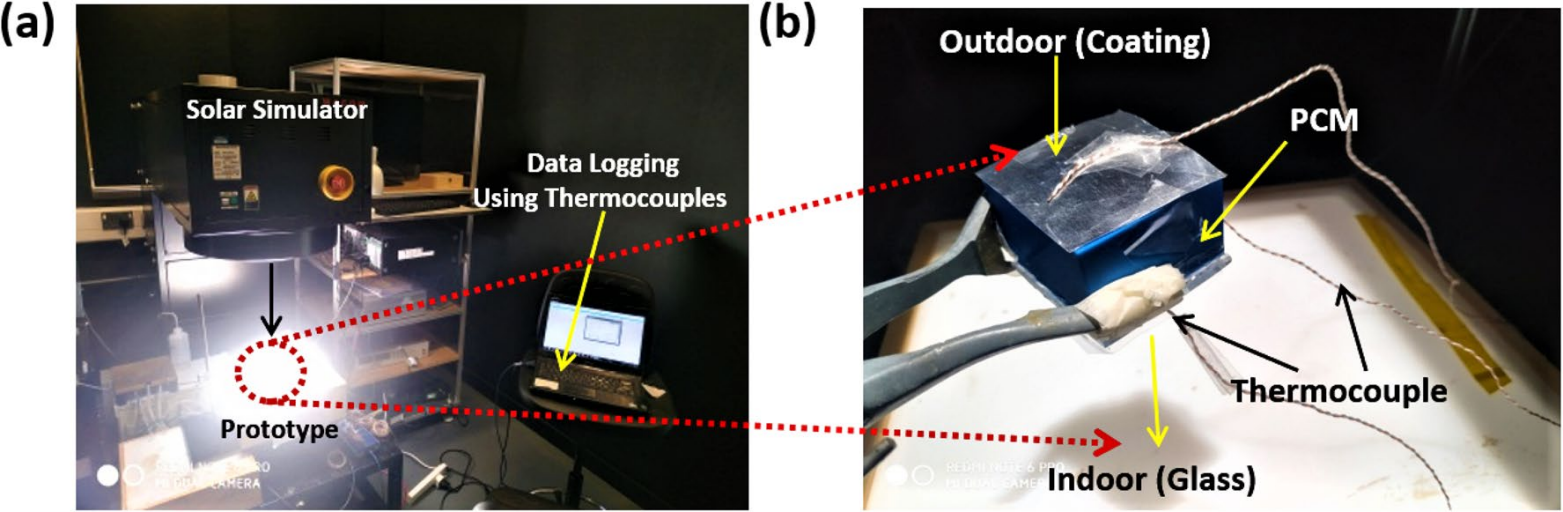
Photograph of the (**a**) overall experimental set-up using GOC based prototype chamber under 1 SUN 1.5 AM condition, (**b**) testing set up for the temperature measurements across the different part of the prototype chamber, respectively.

The temperature measurements of each component have been measured using the thermocouples under 1SUN 1.5 AM. The temperature difference between top (outdoor) and bottom (indoor) exterior surface side of the prototype chamber has been denoted as ΔT. The photographs of the overall testing process have been described, as shown in Fig. 2a. Further, Fig. 2b represents the testing set up for the temperature measurements across the different part of the prototype chamber.

**Figure 3:**
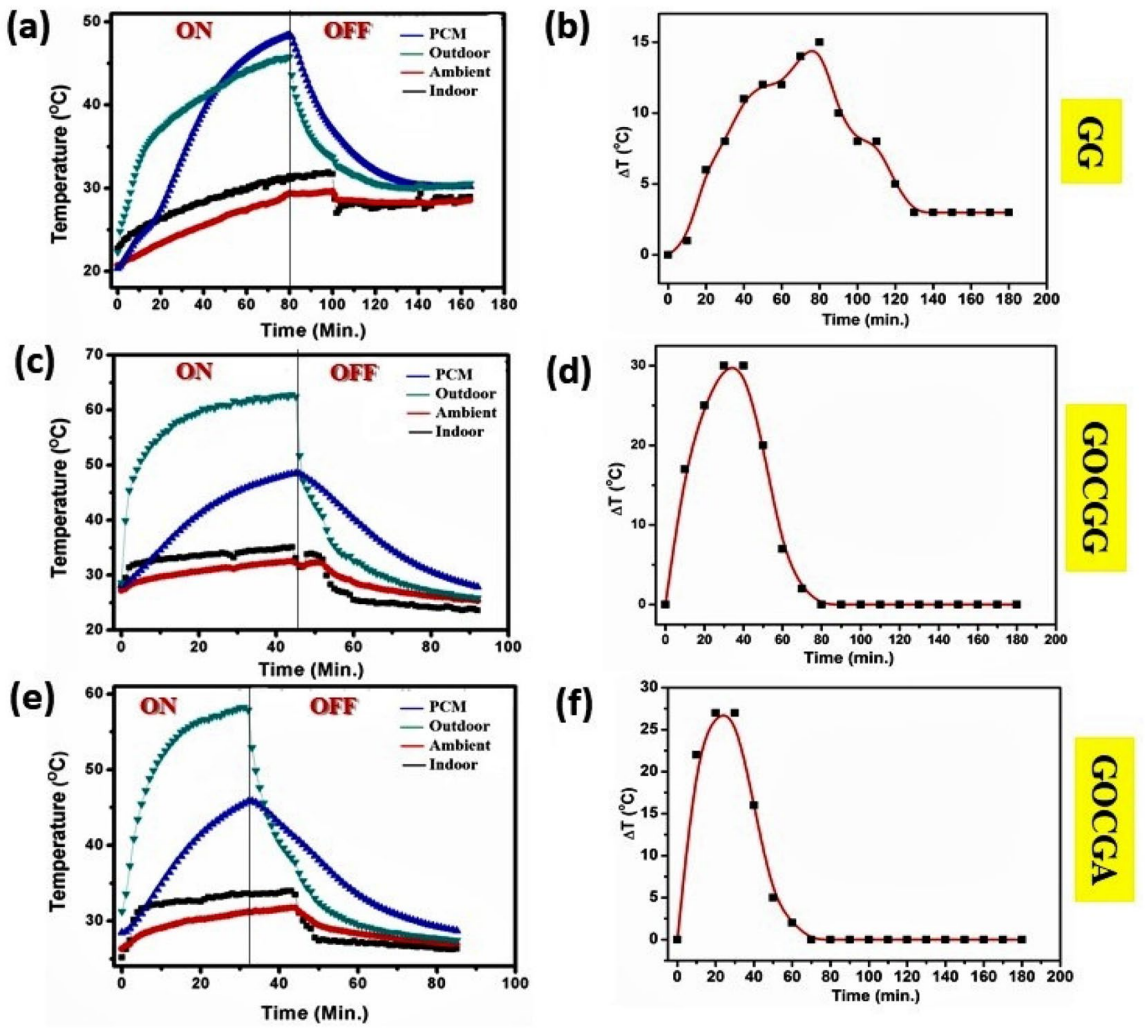
Temperature profile plot of (**a**) GG, (**c**) GOCGG, (**e**) GOCGA system and their corresponding ΔT plot in (**b**), (**d**), (**f**), respectively.

In the case of the GG system, the effect of GOC was quite impressive to enhance and modify the indoor temperature as observed from Fig. 3. In the absence of GOC, the heat transmitted to the PCM and therefore resulted in a lower indoor temperature, as shown in Fig. 3a. Corresponding ΔT profile has been given in Fig. 3b, which shows a maximum ΔT value of ~ 15 °C. It indicates the lower temperature difference between indoor and outdoor, which further signifies the low thermal insulating building envelope. Whereas, in the case of GOCGG, the introduction of GOC successively maintained higher indoor temperature and further able to hold on the temperature ≥ 32 °C (Fig. 3c). In this case, the results further correspond to the ΔT profile, which indicates the maximum temperature difference lifted significantly to 30 °C as shown in Fig. 3d. Higher temperature difference indicates improved thermal insulation compared to Fig. 3a.

On the other hand, replacement of indoor glass substrate by Al in a case of GOCGA resulted in the same phenomena as observed for GOCGG as shown in Fig. 3e. However, the ΔT profile exhibits a maximum temperature difference of ~ 27 °C (Fig. 3f). This is because of the higher thermal conductivity (~ 205 W m^−1^ K^−1^) and emissivity (0.09 -0.1) of Al compared to glass^49^. Besides, during the cooling time, the GOC systems exhibit faster temperature reduction compared to without GOC system. This is because GO has a higher emissivity and a larger surface area than Al^50^. It is, therefore, anticipated that the generated heat escapes to the exterior of the GOC through the pores of GO and facilitates the faster heat dissipation. Not only that, but the GOC system also diminishes the ΔT in such a way that it can be minimized and retain this for a long time as observed up to 180 min. Therefore, for the glass-based outdoor cover, the maximum ΔT trend follows as GG <  < GOCGA < GOCGG. Whereas, the minimum ΔT trend exhibits as GG <  < GOCGA ⁓ GOCGG. For all the cases, it has been observed that the temperature drops down curves follow an asymptotic trend after reached the maximum temperature saturation.

**Figure 4:**
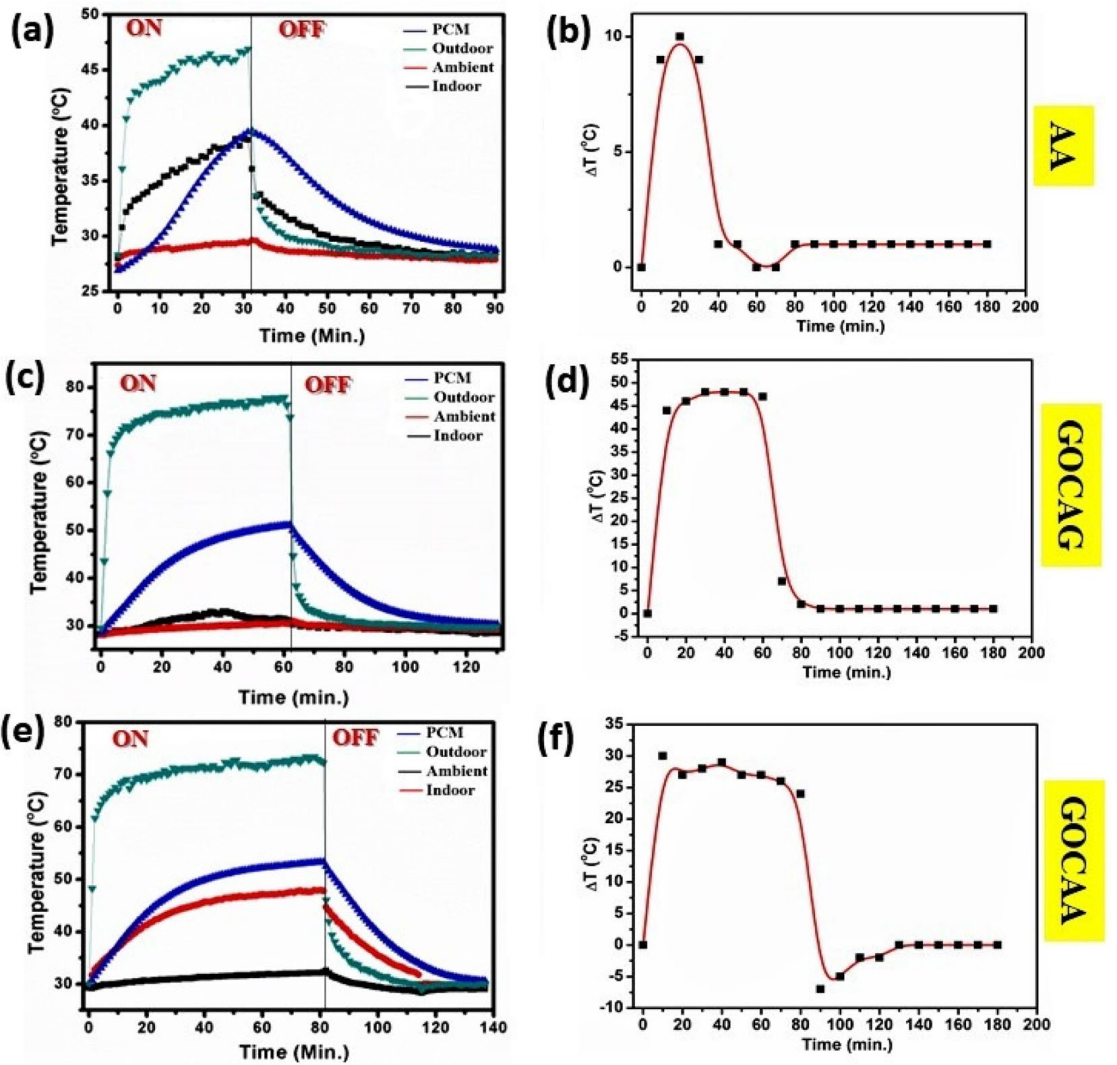
Temperature profile plot of (**a**) AA, (**c**) GOCAG, (**e**) GOCAA system and their corresponding ΔT plot in (**b**), (**d**), (**f**), respectively.

The effect of GOC as a high temperature resistant has also been executed for Al-based substrate, as shown in Fig. 4. Al itself a conducting metal and therefore allows more temperature and leading to rising the exterior surface temperature up to 45 °C as shown in Fig. 4a in case of AA system. On the other hand, the indoor temperature rises to ~ 38 °C (Fig. 4a). As a result, the corresponding ΔT profile indicates a shallow maximum temperature difference about 10 °C, as shown in Fig. 4b. This indicates in the absence of GOC, thermal insulation across the exterior and interior is low.

Interestingly, the exterior temperature was reduced to 25 °C in case of using the GOC on Al substrate for the GOCAG system, as shown in Fig. 4c. The maximum ΔT was recorded for GOCAG of ~ 48 °C (Fig. 4d), which indicates higher insulation. On the other hand, the indoor temperature was enormously increased to ~ 45 °C in case of GOCAA system. However, the maximum outdoor temperature upraised to ~ 45 °C, and resulted in a maximum ΔT of ~ 31 °C, as shown in Fig. 4e,f. It is noted here that the temperature rises to 70 °C within 10 min.

For all the cases of Al, it has been observed that during the cooling period, the temperature drop down obeys relatively faster reduction trend compared to the glass-based system. Besides, during the cooling time of outdoor temperature, it almost reaches to ambient as well as indoor temperature in case of GOCAG and thus minimized the ΔT. However, the ΔT minimization does not follow any trend for AA and GOCAA system. Even, in the case of GOCAA, the ΔT value reaches negative also. Therefore, the results exhibit the maximum ΔT fashion as GOCAA <  < AA < GOCAG. Whereas, the minimum ΔT follows a trend as GOCAA <  < AA <  <  GOCAG.

The temperature profile for both glass and Al-based GOC reflects the effect of GOC during its exposure to the sun and further cooling period. In both cases, using glass-based indoor substrate reveals better performance compare to Al-based indoor substrate in terms of both maximum and minimum ΔT parameter. Also, for both the cases, the minimum ΔT has been reduced to almost zero, and this has been further maintained for a long time as obtained up to 180 min. As a result, this observation shows quite impressive performance of GOC as a head spreader to diminish the indoor temperature and further able to have appeared as a passive heating strategy. Also, the temperature reduction further enfolds high efficiency in case of the glass-based indoor substrate. It has been observed that Al-based substrates are light weighted and flexible compared to the glass-based substrate and therefore, excels priority to use. However, as Al itself, a conductive metal allowed more heat and resulted in comparatively higher outdoor temperature than glass. Beginning with the preliminary planning and factoring in the design of the GOC, timely identification of any coating formulation, architectural or fabrication defects that will compromise the coating such as instability to long term heat, water-resistant, flexibility, structural defects etc. To further understand the coting stability and GO structural effect for different temperature and water treatment, studies on hydrophobic, flexible and high-temperature resistant GOC has been further performed.

### X-ray diffraction and Raman spectroscopy studies of GOC for different temperature treatment

**Figure 5:**
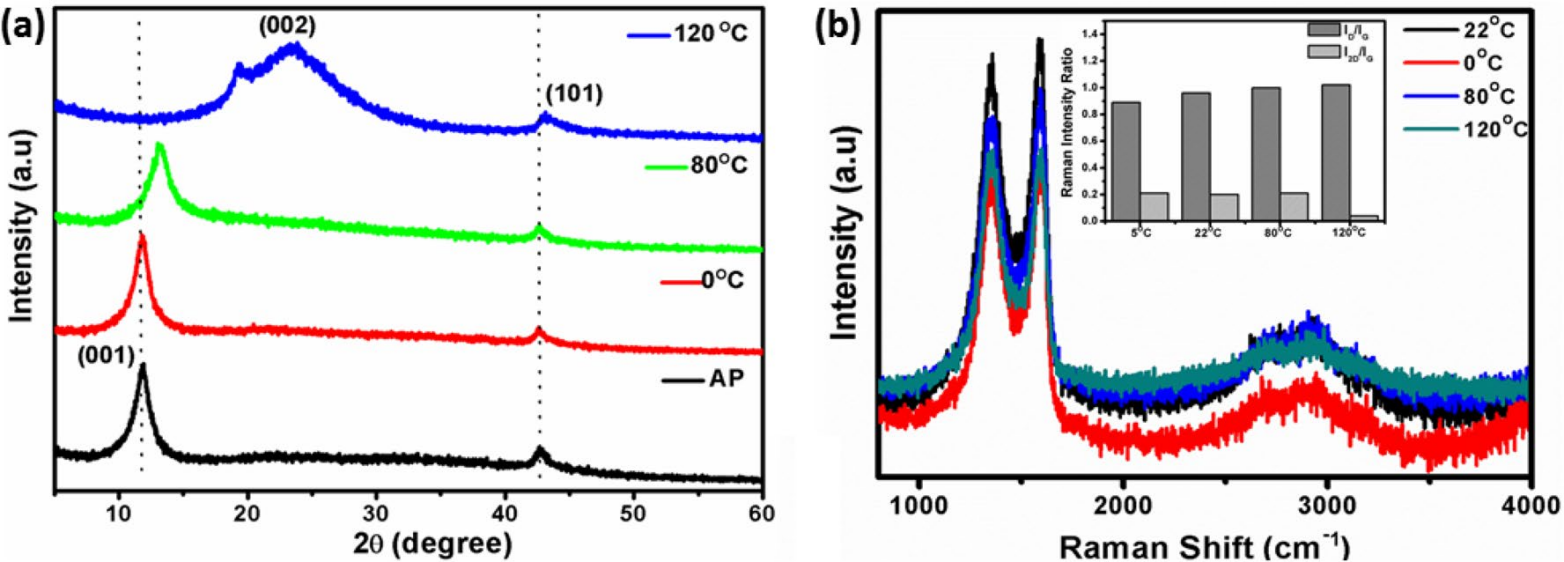
(**a**) X-ray diffraction pattern and (**b**) Raman spectra (Inset: Raman intensity ratio plot) for 0, 22 (as prepared: AP), 80 and 120 °C temperature treated GOCs, respectively.

The XRD pattern of the four different temperature treated GOCs, as described in the experimental section was shown in Fig. 5a. A sharp reflection ~ 12.4^o^ and a weak reflection ~ 44.5°, respectively, were observed for the as-prepared GOC sample. The diffraction peaks correspond to the (001) and (101) planes of the graphene oxide. Moreover, as shown in Fig. 5a, during the temperature variation, the (001) peak disappears while the (002) peak shifts to the position, which is a typical characteristic for reduced graphene oxide (~ 24.6°). Besides, the (002) peak does not maintain its original intensity and width, which points to a degree of reduced graphene oxide exfoliation as observed at 120 °C. Besides, above 120 °C the (002) peak does not exhibit significant changes, either in position or in width, which suggests that the graphene layers in the treated materials have comparable interlayer spacing and similar stacking thicknesses. However, the (001) peak of the coating obtained at 80 °C is some-what broader. It appears at slightly higher 2θ values, indicating that some intercalation is still present due to the dangling bonds and less water and/or labile oxygen-containing groups presence. The XRD results suggest the coating can be extensively stable up to 80 °C, a typical harsh hot temperature for the environment.

The Raman spectra of the GOCs for different temperature is featured in Fig. 5b. The characteristics Raman bands observed at 1354 cm^−1^ (D- band), 1582 cm^−1^ (G-band), and 2712 cm^−1^ (2D -band) in case of the GOC sample. Typically, the single-crystal graphite sp^2^ carbon- framework (E_2g_ mode) represents the G-band. On the other hand, D band (A_1g_ mode) signifies the presence of nanocrystalline graphitic orientation originated from the sp^2^ framework disorderliness by forming sp^3^ bonds^51^. In this case, it has been observed that the G band ~ 1596 cm^−1^ and D band ~ 1348 cm^−1^ thereby confirming the existence of nanocrystalline graphitic sp^2^ clusters. The presence of G and D-band is observed for all the different temperature treated coatings samples, but interestingly the D-bands are broadened, and G-bands become more intense at 22 °C coating sample. The intensity ratio between D, G and 2D peaks has been used to evaluate the graphitization of the graphene oxide coated samples (Inset: Fig. 5b). The effect of high temperature increases the I_D_/I_G_ ratios from 0.889 to 0.987, indicating a high degree of graphitization. This ratio is also a measure of the degree of disorder and sp^3^/sp^2^ carbon atoms. There is also a stark difference in the full width at half maxima (FWHM) of both G and D bands of the coating samples. This shortening of FWHM for different temperature is a direct indication of an increased level graphitization order due to the release of the presence of sp^3^ carbon and an increase in the graphitic domain size^51^. This was also evident in the XRD patterns of the different temperature treated coatings (Fig. 5a) where the reduced graphitic carbon zone is considerably broadened due to high temperature. The same intensity of the D band also implies a decreased level of disorder and an increase in the graphitic domain size. The recombination of graphene layers at high temperature introduces higher order of graphitic domains with lack of orderings. This could again be a clear indication of the formation of disordered combined graphitic layers^51^.

### Microstructural investigation of GOC

**Figure 6:**
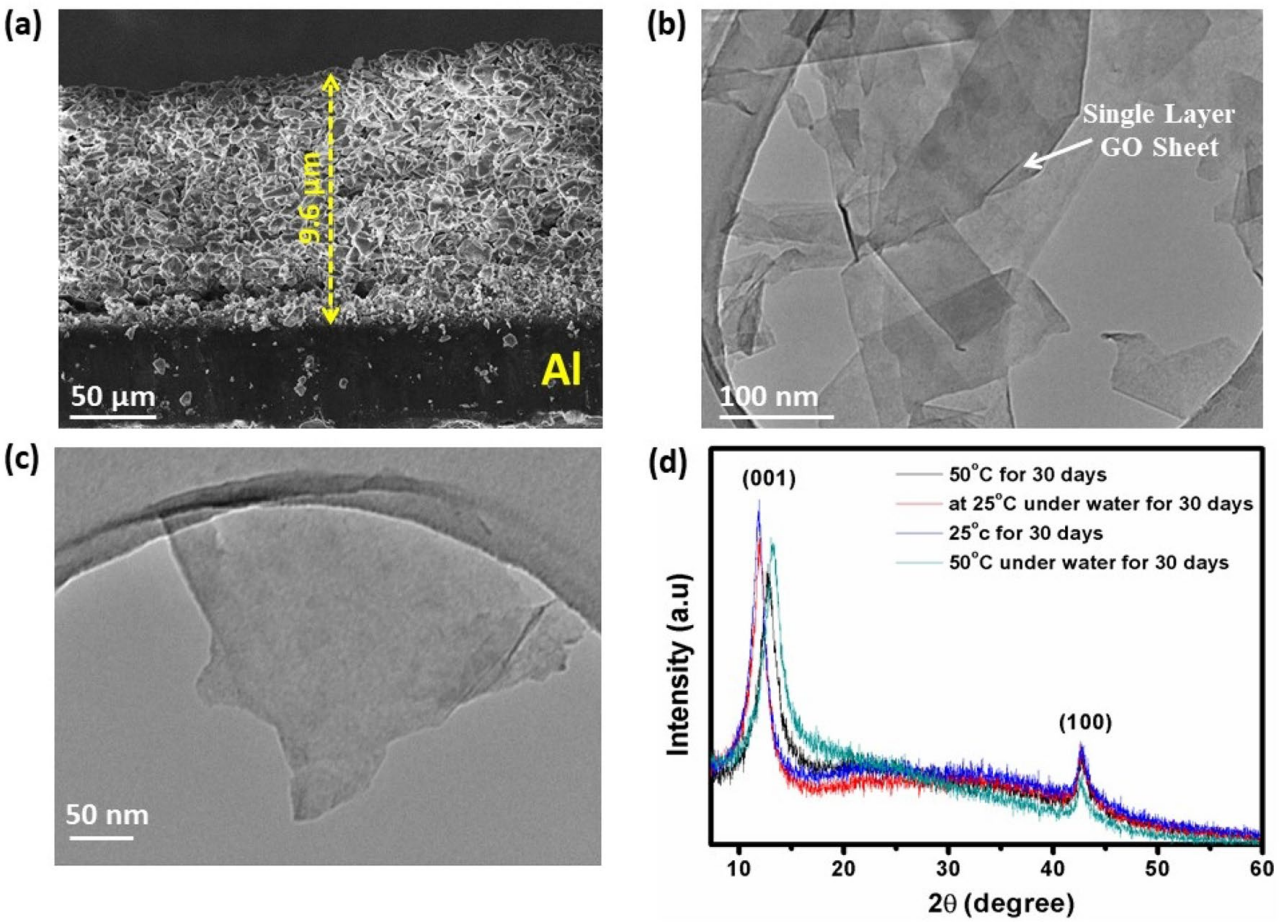
(**a**) SEM microstructure image of GOC on Al; TEM bright-field images for the GO sample at (**b**) lower, (**c**) higher magnification, respectively and (**d**) X-ray diffraction pattern of different condition applied GOCs for stability study against temperature and water.

The microstructural image in Fig. 6a manifests the GO layer on Al-sheet and revealing a thickness of ∼9.6 μm indicates that the GO sheet was successfully deposited. Moreover, the microstructure image also indicates that the dimensions are quite uniform as achieved by the screen-printed method. The TEM bright-field images of screen-printed GO sample were recorded at different magnification, as shown in Fig. 6b,c respectively. The bright field exhibits a continuous two-dimensional lamellar structure with wavy folds forms an uneven surface with folds of a certain thickness, and there are apparent wrinkles. This finding confirms the single-layer feature of the graphene oxide sheets. The graphene oxide sheets have lateral dimensions ranging from nano to sub-microns. In order to understand the stability of the developed GOC samples in terms of their thermal and water-resistant effect, the coatings have been further explored towards heat and water treatment together for 30 days (Fig. 6d). The GOC was dipped inside a water bath and maintained the temperature of 50 °C (high) and 25 °C (room temperature) for 30 days, respectively.

Further, to evaluate the structural effect of the coatings, the XRD study of the different conditions applied coating samples are evaluated as shown in Fig. 6d. At high temperature, the (001) plane shifted towards higher 2θ value and at the same time extent of the amorphous region started to develop near the 2θ of 22–24° in case of long-term heat and water treatment affecting to GO. Besides, the TEM bright-field images of the 50 °C and water treated GOC sample for 30 days still exhibits a single-layer feature of the GO sheets, as shown in Fig. S2, SI. It has also been observed that with increasing the heat and water treatment period, the degree of folding in the graphene layers reducing fast. Fig. S2, SI represents the colour is more resonant with thicker graphene layers and shallow when the graphene layer is relatively thin. The TEM study signifies that the GOCs are quite consolidating and long-lasting for heat and water treatment as monitored up to 30 days.

### Investigation of GOC stability towards water and heat treatments

**Figure 7:**
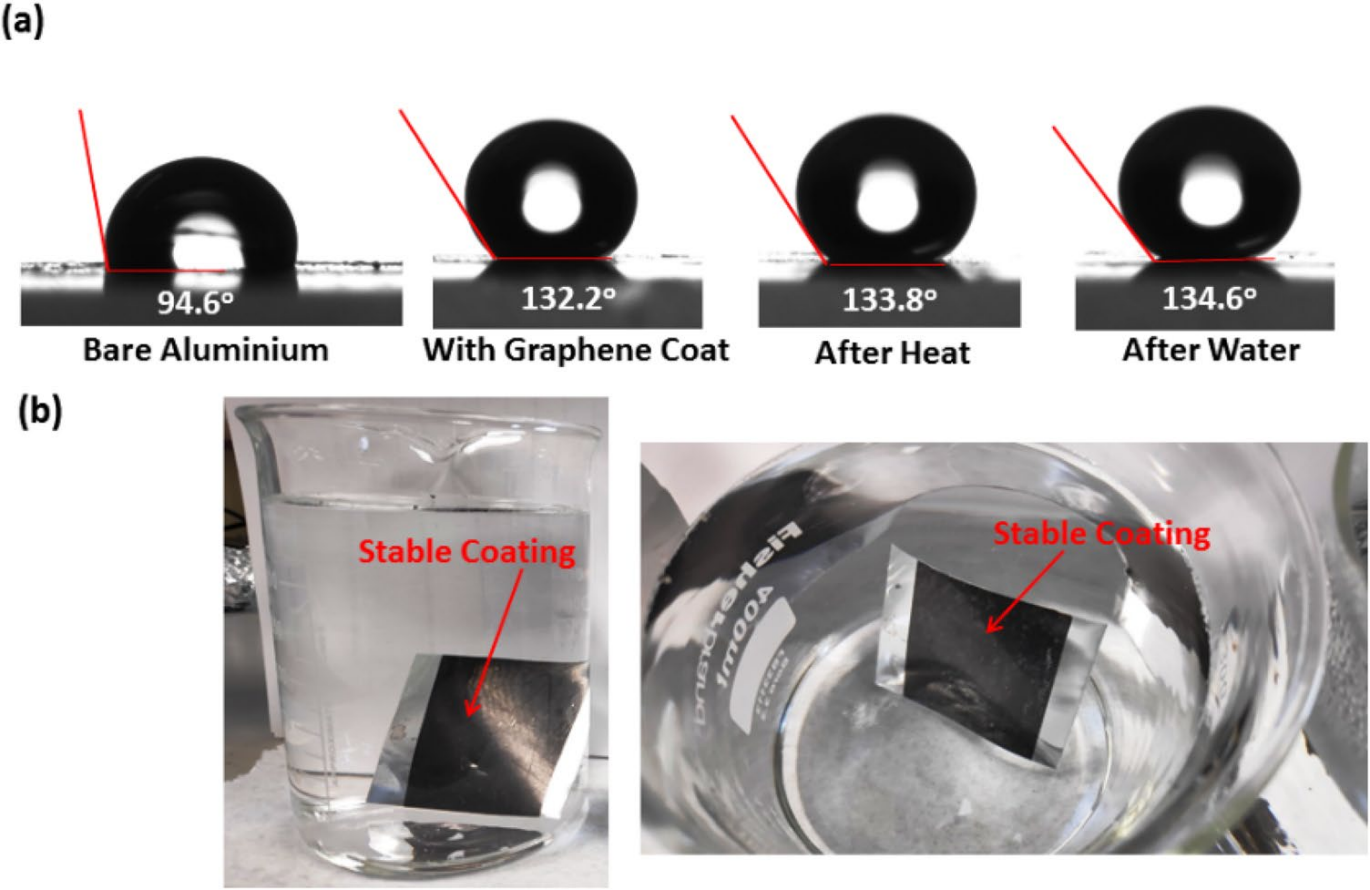
(**a**) Water contact angle measurement profiles of different condition applied GOCs and (**b**) digital photographs of the water-resistant GOC.

Fig. 7a presented the water contact angle (WCA) measurement of the as-fabricated GOC and its allowed treatment for 50 °C temperature and water dipping treatment for 30 days, respectively and compared with the bare Al substrate. The as-prepared GOC exhibits a hydrophobic WCA of 132.3 ± 1.0°. Interestingly, after the constant 50 °C temperature and water dipping treatment for 30 days, the same GOC sample maintained its hydrophobic character exhibiting a WCA of 133.8 ± 0.8° and 134.6 ± 1.0°, respectively. It is assumed that the microstructure of the GO consists two-dimensional lamellar morphology with wavy folds, which provided sufficient "air pockets", thus enabled water droplets to bounce and roll off immediately as consistent with Cassie-Baxter model^52^. The model demonstrates that the trapped air may serve as air cushions, providing a resistance to the penetration of water droplets to the GOC. The recorded WCA images of the GOC inside regular tap water represents the stability of the coating. Hydrophobic behaviour character is also perceived from the WCA analysis, as shown in Fig. 7b.

### Electrical resistance and conductivity of GOC

**Figure 8:**
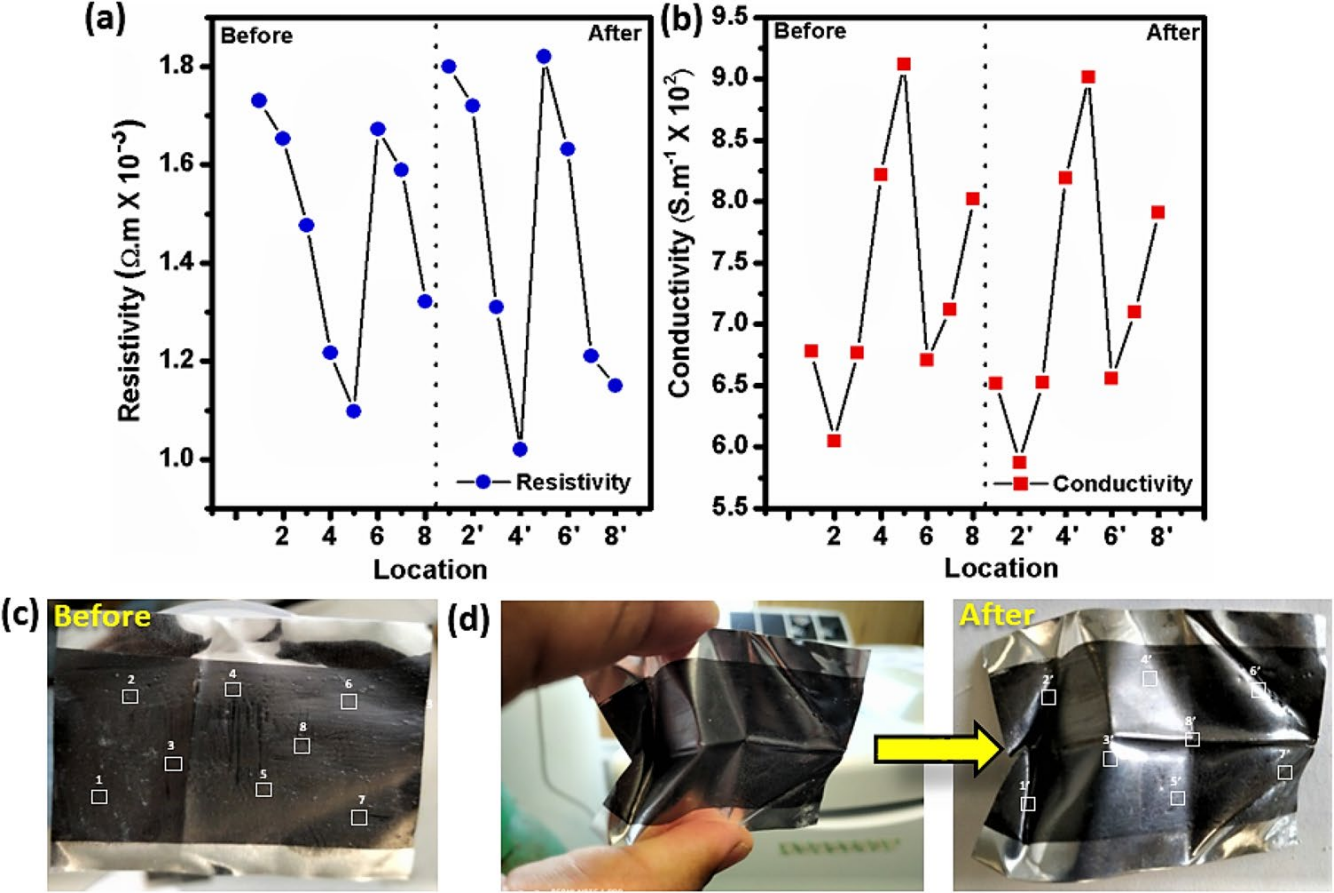
(**a**) Resistivity, (**b**) conductivity measurement plot of the GOC as obtained from different locations (marked) in the digital photograph of (**c**) as prepared (before) and (**d**) after squeezing of GOC, respectively. (White boxes indicate the location where the resistivity and conductivity measurements have been performed, and prime numbers designate about the deformed location after squeezing of GOC).

Electrical conductivity and robust mechanical property of the GOC promises its application for flexible material. To investigate the electrical resistance and conductivity performance of GOC under squeezing condition, the four-probe measurements have been performed accordingly, as shown in Fig. 8a,b, respectively. The resistance and conductivity measurements have been performed in randomly selected eight different locations as designated in Fig. 8c. Interestingly, even both the resistance and conductivity property of the GOC remains unaffected after the squeezing condition (Fig. 8d). This result interprets about the flexibility of the GOC, which excels its mechanical robustness, substantial flexibility and a higher degree of toughness resulting to appear as a stable coating. The digital pictures, as shown in Fig. 8c,d designates about the random squeezing of the GOC, exhibits almost equivalent resistant and conductivity compared to its original form as shown in Fig. 8d, respectively.

### Thermal image analysis of GOC

**Figure 9:**
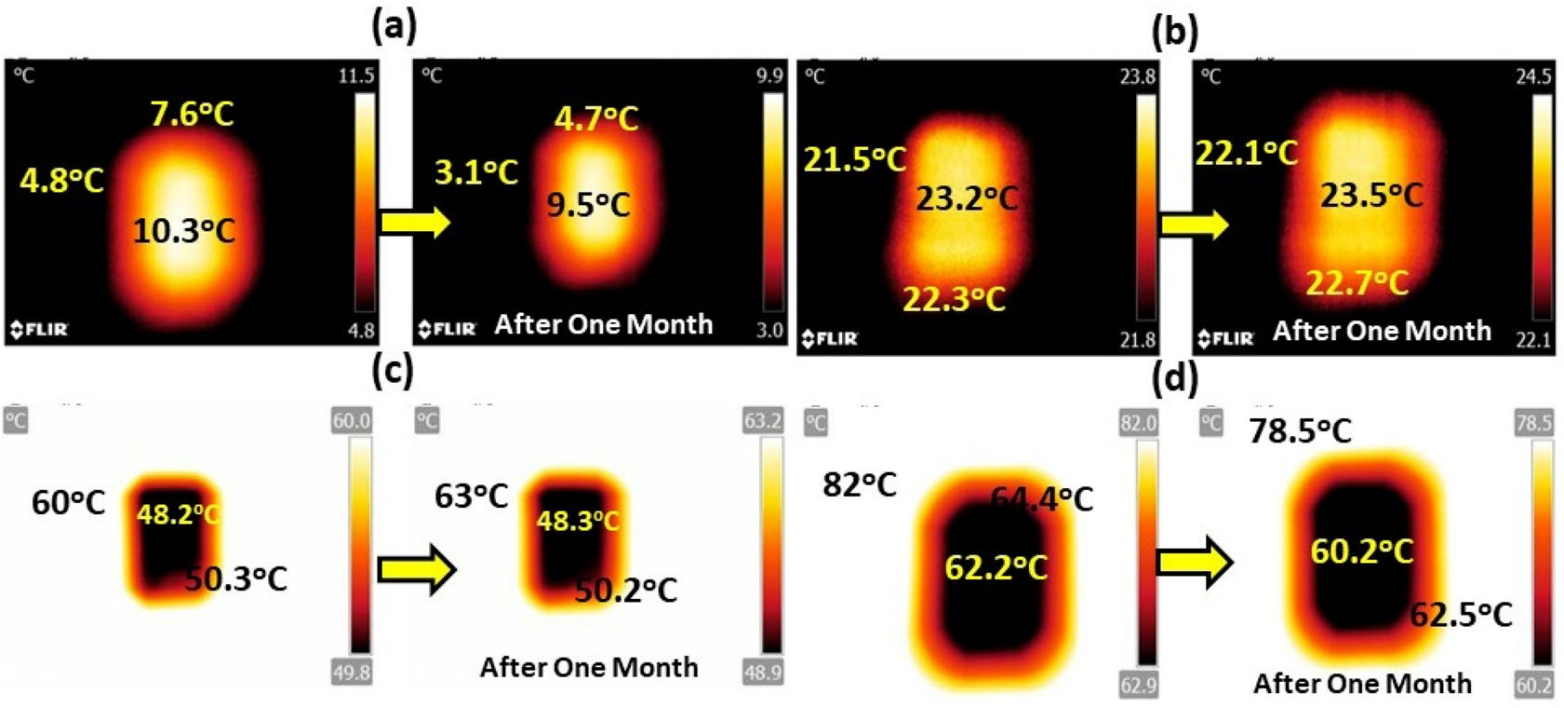
FT-IR thermal image analysis of GOCs at (**a**) lower (4 °C), (**b**) room (22 °C), (**c**) high (60 °C), and (**d**) very high (80 °C) temperature zone in terms of natural weather for instant and after a month record, respectively.

Now, to evaluate the temperature treatment effect of the developed GOC, the images for different temperature applied condition for 30 days using infra-red (IR) thermal image camera was recorded, as shown in Fig. 9a–d. The IR camera shots were taken with a FLIR T425 camera positioned top of the coating and monitored the image for a long-range of temperature window starting from 3 to 80 °C. In Fig. 9a, it has been noticed that the GOC part consists higher temperature compared (10.3 °C) to the outside temperature (4.8 °C), which allows higher order of thermal comfort as received from the graphene oxide. Further, this performance has been monitored up to one month, keeping the same thermal difference.

Interestingly, at room temperature, the temperature difference between outside and the coating surface was found considerably insignificant (Fig. 9b). Furthermore, the temperature difference upraises in case of higher applied temperature (outside). The difference is ~ 9.7 °C, as shown in Fig. 9c, and the difference enhances to 20 °C when the outside temperature rises to 82 °C, as shown in Fig. 9c,d. Appraisal of these IR thermal images reflects indication about the effective use of GOCs, which can able to maintain the comfortable indoor temperature at a colder or hotter outside temperature and resulted to a promising thermal comfort candidate.

### Probable mechanism

We suggest an apparent mechanism to understand the advantages of these GOCs. It is anticipated that due to consist of flat electronic two dimensional band structure, GO evinces an exceptional horizontal heat conductor with high thermal stability as solar absorber coatings compared to the conventional solar absorber coatings. Typically, the large extent of sp^2^ bonds density excel a remarkable capture of the electrons during the diffusion and oxidation process which makes a broad Brillouin zone of the band structure of the GO^53–55^. GO absorbs and scatters photons at the upper and then a part of photons is incident on the indoor side. For instance, GO benefits from the high *in-plane* thermal conductivity, up to a particular channel length. However, weak thermal properties for the substrates implies that interfaces and contacts remain the significant dissipation bottlenecks. The heat generation mainly originated from the phonon–phonon coupling within the graphene electronic band structure. Despite that the molecular dynamics method interprets about the sp^2^ hybridized bonded orbitals of graphene and its derivate structures, generating extremely stiff links between atoms^56^. This stiff bond is at the origin of too high, and maybe diverging, the thermal conductivity of graphene and thus leading as an efficient thermal barrier^57^. The stoichiometric oxygen coverage introduces dramatic structural deformations, which signifies the thermal conductivity of the GO^58^. In other words, GO does not collapse the underlying hexagonal lattice frame instead introduces local strain in the lattice symmetry of carbon atoms and integrity, thus disturb the thermal transport weakly. Thermal decomposition (in the ambient atmosphere) at higher temperatures was reported to result in a highly disordered mixture of various oxygen-containing graphitic carbons that are difficult to characterize^59^. In order to decrease the temperature further; drastic quality improvement of the graphene film is required and can be opt-out for future research interest.

The quality and thickness of the printed layers depend on the rheology of the paste too, so it can be stated that it is possible to select of the optimal composition of the paste to improve its printability. Further, the simplicity of the GO paste preparation on a large scale and its high sensitivity towards temperature reduction makes it convenient for practical application. This work also provides insights into the design of the graphene-based coatings to remove the heat from the buildings’ roof. The obtained results are quite encouraging and open up ample scope to develop new and economically viable synthetic approaches for enhancing performance as the thermal comfort material and could be projected as "wallpaper" for building roofs. However, issues like undesirable optical loss large-scale; production of planar patterned structures; better adhesiveness; and cost-effectivity needed to be addressed before achieving a graphene-based perfect absorption structure.

## Conclusions

A novel graphene oxide coating (GOC) has been developed by screen-printing technique, which plays a crucial role in improving the thermal comfort and thermal stability along with the water-resistance performance. The overall experiment has been executed using a 3D printed prototype chamber, where GOC has been used as a top cover on the low iron glass or aluminium-based substrate (5 × 5 cm^2^) connecting through a phase change material channel in contact with direct sun exposure. Using GOC on Al sheet resulted in a maximum temperature difference (ΔT) of ~ 50 °C, while GOC on glass substrate offered the maximum ΔT increment to ~ 30 °C. Besides, the reduced temperature was maintained for a more extended period compared to the outside temperature. The experimental results showed promising thermal comfort behaviour of the proposed material. Further, the stability of GOC has been extensively monitored at different temperature and towards the water. Two crucial microstructural characterizations techniques of graphene oxide such as X-ray diffraction and Raman spectra analysis further signified about the physicochemical properties of the different temperature treated GOC. Despite that, the water contact angle measurements established the hydrophobic behaviour of the GOC, which shows water-repellent characteristics of the coating. Besides, GOC exhibits a very high order of mechanical robustness, substantial flexibility and a higher degree of toughness. All these characterization leads to develop a stable coating of graphene oxide. Thus, the outcome from this work is highly promising and renders a potential for future passive cooling and heat sink tandem structure concomitantly. The results will trigger research furthermore as well as develop more commercial applications in this area.

## Materials and methods

### Synthesis of GO paste for screen-printing

The graphene oxide (GO) powder was purchased from SIGMA ALDRICH (796,034) and employed without any further purification. 1 g of GO powder (12 mg mL^−1^ dispersion) was mixed with 120 mL of isopropanol (MERCK) and 3 g of a 3:2 copolymer of N -vinyl-2-pyrrolidone (NMP, SIGMA ALDRICH) and vinyl acetate (PLASDONE S-630, ASHLAND INC., USA) to constitute GO: binder weight ratio of 1:3, and obtained a thick black slurry. The thick slurry was further sonicated using a stainless steel-based probe sonicator for 1 h. This was carried out under constant cooling with iced water, within a few minutes of mixing at 2000 rpm the slurry converts into a homogeneous dispersion, which is then subjected to treatment at 5000 rpm. This dispersion was passed through a 3.1 μm glass fiber filter membrane (ACRODISC)^60^. Further, the dispersion was mixed with 1.5 g ethyl cellulose (SIGMA ALDRICH) in 10 ml of ethanol. After adding 1 mL terpinol (SIGMA ALDRICH) to the blend, it was stirred thoroughly and subsequently dried off the ethanol from the solution in a vacuum oven to obtain a viscous GO paste.

### Development of screen-printed GOC

**Figure 10:**
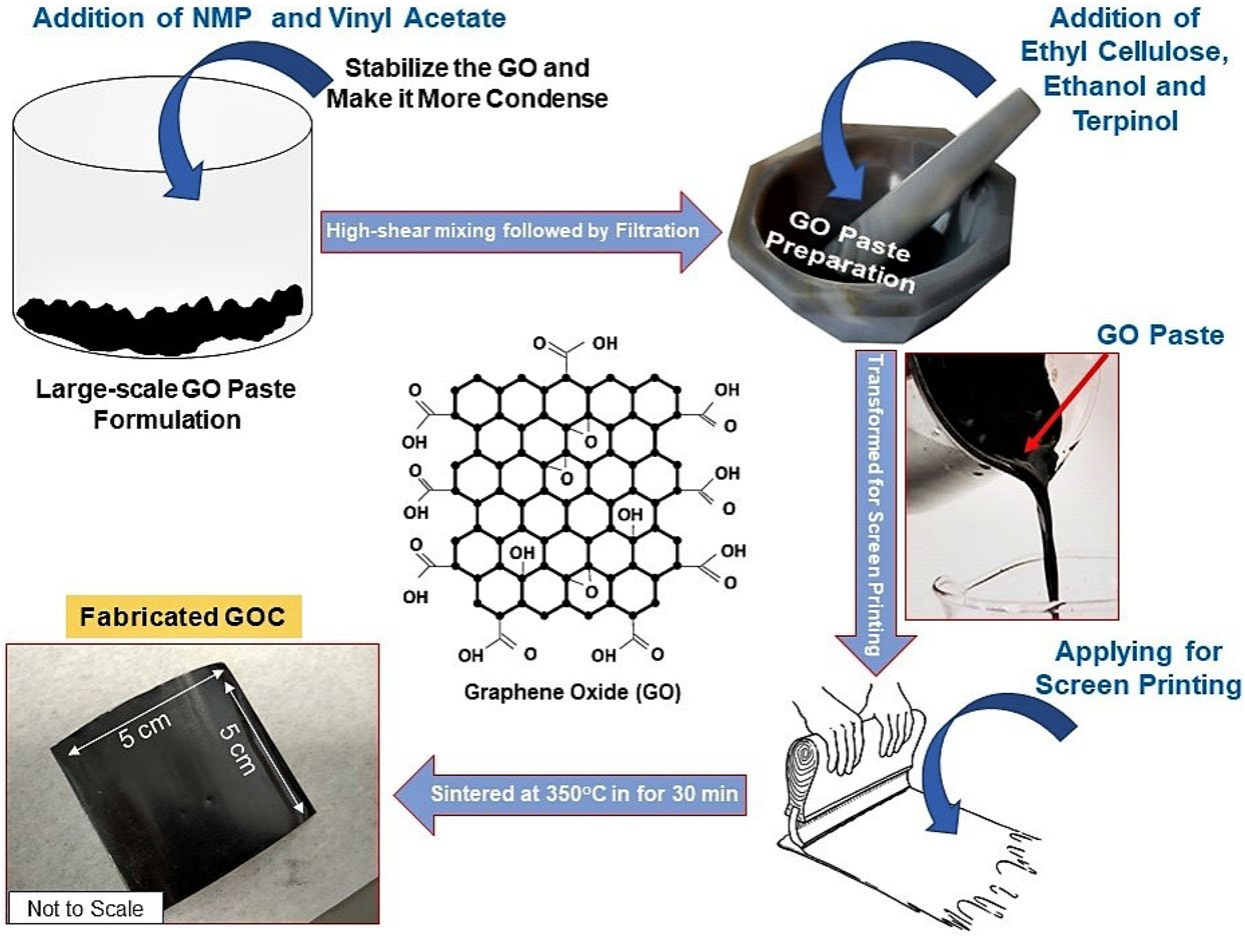
Schematic of GO paste preparation and screen-printed GOC fabrication process.

A layer of as-prepared GO paste was employed to develop the coating on a 5 × 5 cm^2^ Aluminium (Al) sheet (SIGMA ALDRICH, Z740226) and low-iron glass by screen-printing (120 T mesh/inch, MASCOPRINT, UK) method, respectively**. **The prepared coating was then sintered in the hot plate at 350 °C for 30 min to remove the binders and designated as graphene oxide coating (GOC) for further studies. Fig. 10 describes the paste preparation followed by screen-printed coating process schematically.

### Thermal characterization of as-prepared paste

The decomposition behaviour of as-prepared GO paste was investigated by thermogravimetric analysis (TGA). Fig. S1, SI shows the thermal stability of the GO paste, which is relatively good. However, at a temperature of about 200 °C, the thermal stability of GO was found to decrease due to the decomposition of carboxylic and release of CO_2_ gas.

### Experimental set-up

In a 5 cm × 2 cm × 2 cm 3D-printed prototype chamber (aperture area: 0.48 m^2^), 10 g of PCM has been kept in between the outdoor and indoor substrates. This system is leakage proofed and lightweight, too (Fig. 1). Throughout this experiment, PCM, RT 25 as purchased from RUBITHERM GmbH has been used without further modification. The PCM has been selected as per their melting temperature during the experiment. As soon as the PCM has entirely changed its phase from solid to liquid and upon further heating, the temperature lifts again, as expected. Despite that, to understand the temperature effect on GOCs, they were placed under 0, 22 (considered as room temperature), 80 and 120 °C heat chamber for 30 h, respectively. The selected temperatures suggested about the different weather temperature conditions such as 0 °C as low, 22 °C as room temperature followed by 80 and 120 °C as high temperature during the experiment. Besides, the GOC samples were immersed under tap water at 50 °C temperature to monitor the effect of both temperature and water on the coating.

### Material characterization

Thermogravimetric and differential thermal analysis (TG–DTA) of the as-prepared paste was carried out from 30 to 600 °C at a heating rate of 10 °C min^−1^ (DSC 214 Polyma from NETZSCH) to understand the thermal decomposition characteristics. X-ray diffraction (XRD) analysis were characterized by BRUKER D8 Advance X-ray diffractometer (Cu Kα irradiation, 40 kV/40 mA, 0.02° 2θ step size and a scan time of 3 s per step) in the range of 5–60°. The cross-sectional microstructural SEM image of the coated sample was analysed on a TESCAN VEGA3 SEM. The morphology of the GO sample was characterized using a transmission electron microscope (TEM) (HITACHI S3200N SEM, Tokyo, Japan)^56^. Raman spectroscopy was undertaken using a HORIBA Jobin Yvon LabRAM HR Raman Spectrophotometer, Kyoto, Japan (with 632.8 nm He–Ne laser). The spectrum was recorded in the range of 100–1000 cm^−1^. The relative surface wettability of the films was accomplished by measuring the successive contact angle measurements (goniometer from OSSILA, U.K). The resistivity and conductivity measurements were performed using the Four-Point Probe Instrument by OSSILA, U.K. The infra-red (IR) images were taken with a FLIR T425 camera positioned on top of the GOCs at the base by 10 mm. The temperature profile of the prototype chamber was measured under 1000 W m^−2^ (1 SUN 1.5 AM) of light from a WACOM AAA + continuous solar simulator (model WXS-210S-20)^56^. Temperature recording was performed using the TC-08 thermocouple data logger (PICO TECHNOLOGY).

## Supplementary information

Supplementary Information.
