# Supplementary material for: Emplacement of screen-printed graphene oxide coating for building thermal comfort discernment
Source: Sci Rep. 2020 Sep 23;10:15578. doi: 10.1038/s41598-020-72670-8 (PMC7511961; doi:10.1038/s41598-020-72670-8)
Supplement: Supplementary file 1 — Supplementary Information. [file 41598_2020_72670_MOESM1_ESM.docx]

**Supplementary Information**

**Emplacement of screen-printed graphene oxide coating for building thermal comfort discernment**

Anurag Roy,* Aritra Ghosh, David Benson, Tapas Kumar Mallick and Senthilarasu Sundaram*

Environment and Sustainability Institute, University of Exeter, Penryn Campus, Cornwall TR10 9FE, U.K.

Email: [A.Roy30@exeter.ac.uk](mailto:A.Roy30@exeter.ac.uk) , [S.Sundaram@exeter.ac.uk](mailto:S.Sundaram@exeter.ac.uk)

**Thermal analysis**

In order to find out the stability of the synthesized graphene powder, we carried out simultaneous thermogravimetric-differential thermal analysis (TGA-DTA) studies on the as-derived virgin powder as shown in Figure 1. It is evident from the TGA that the as-collected carbon nanoparticle is stable up to 228 °C and starts oxidizing above 400 °C. The high temperature thermal treatment promoted the partial removal of the labile oxygen-containing functional groups, as proposed in the TGA curve obtained for the lyophilized GO sample.


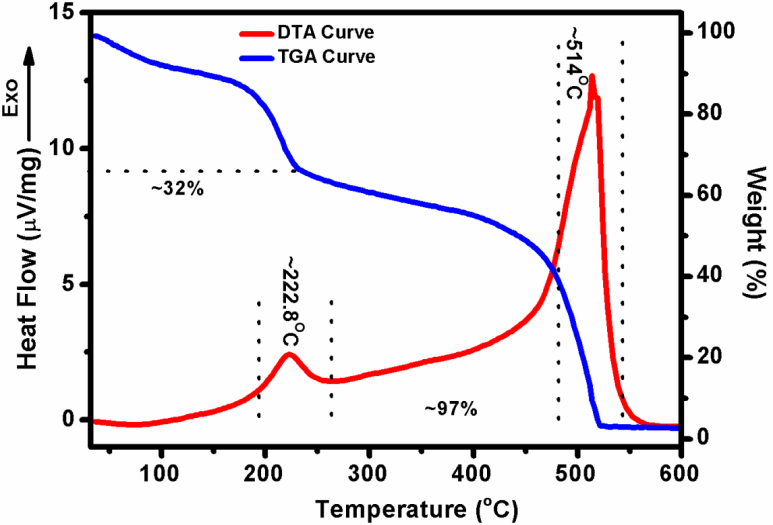


**Figure S1.** Characteristics TG-DTA plot of as-prepared GO paste


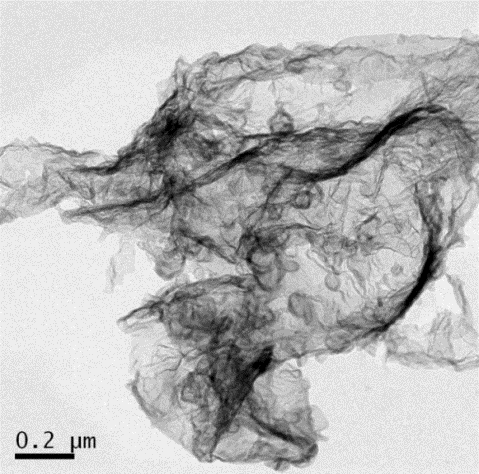

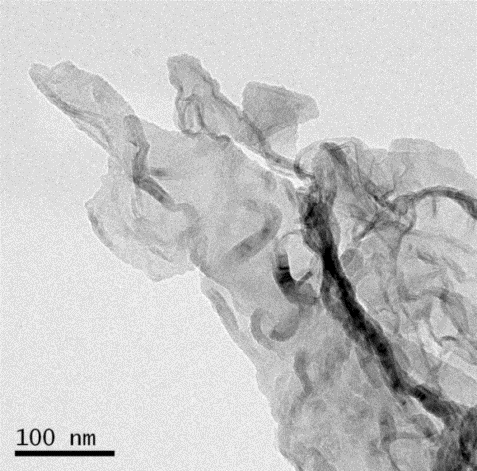


**Figure S2.** TEM bright field image of both 50^o^C and water treated GO sample for a period of 30 days in (i) lower and (ii) higher magnification, respectively.
